# ATZ11 Recognizes Not Only Z-α_1_-Antitrypsin-Polymers and Complexed Forms of Non-Z-α_1_-Antitrypsin but Also the von Willebrand Factor

**DOI:** 10.1371/journal.pone.0091538

**Published:** 2014-03-19

**Authors:** Diane Goltz, Kanishka Hittetiya, Hamideh Yadegari, Julia Driesen, Jutta Kirfel, Thomas Neuhaus, Susanne Steiner, Christiane Esch, Jörg Bedorf, Hans-Jörg Hertfelder, Hans-Peter Fischer

**Affiliations:** 1 Department of Pathology, University Bonn, Bonn, Germany; 2 Institute for Experimental Hematology, University Bonn, Bonn, Germany; 3 Medical Clinic, St. Vincenz-Hospital, Limburg, Germany; Ospedale Pediatrico Bambino Gesu', Italy

## Abstract

**Aims:**

The ATZ11 antibody has been well established for the identification of α_1_-anti-trypsin (AAT) molecule type PiZ (Z-AAT) in blood samples and liver tissue. In this study, we systematically analyzed the antibody for additional binding sites in human tissue.

**Methods and Results:**

Ultrastructural ATZ11 binding was investigated immunoelectron microscopically in human umbilical vein endothelial cells (HUVECs) and in platelets of a healthy individual. Human embryonic kidney (HEK293) cells were transiently transfected with Von Willebrand factor (VWF) and analyzed immunocytochemically using confocal microscopy and SDS-PAGE electrophoresis followed by western blotting (WB). Platelets and serum samples of VWF-competent and VWF-deficient patients were investigated using native PAGE and SDS-PAGE electrophoresis followed by WB. The specificity of the ATZ11 reaction was tested immunohistochemically by extensive antibody-mediated blocking of AAT- and VWF-antigens.

ATZ11-positive epitopes could be detected in Weibel-Palade bodies (WPBs) of HUVECs and α-granules of platelets. ATZ11 stains pseudo-WBP containing recombinant wild-type VWF (rVWF-WT) in HEK293 cells. In SDS-PAGE electrophoresis followed by WB, anti-VWF and ATZ11 both identified rVWF-WT. However, neither rVWF-WT-multimers, human VWF-multimers, nor serum proteins of VWF-deficient patients were detected using ATZ11 by WB, whereas anti-VWF antibody (anti-VWF) detected rVWF-WT-multimers as well as human VWF-multimers. In human tissue specimens, AAT-antigen blockade using anti-AAT antibody abolished ATZ11 staining of Z-AAT in a heterozygous AAT-deficient patient, whereas VWF-antigen blockade using anti-VWF abolished ATZ11 staining of endothelial cells and megakaryocytes.

**Conclusions:**

ATZ11 reacts with cellular bound and denatured rVWF-WT and human VWF as shown using immunocytochemistry and subsequent confocal imaging, immunoelectron microscopy, SDS-PAGE and WB, and immunohistology. These immunoreactions are independent of the binding of Z-AAT-molecules and non-Z-AAT complexes.

## Introduction

Enzyme-linked immunosorbent essay (ELISA) for AAT deficiency type PiZ based on ATZ11 was introduced in 1984 [1]. It was further developed by Wieslab AB (Lund, Sweden) based on the observation that ATZ11 specifically detects a conformation-dependent neo-epitope of both polymerized and elastase-complexed molecular forms of AAT [2], [3]. Because *PiMM* (non-Z) individuals have scarce circulating complexed forms of AAT, no cross-reaction occurs in ELISA.

ATZ11 has been established for the immunohistochemical identification of AAT polymers type PiZ in liver tissue [4], [5], [6]. Soon after its introduction, we demonstrated that ATZ11 reacts with endothelial cells of the portal vein in various non-Z specimens [6]. We suggested that this phenomenon was due to a cross-reaction of ATZ11 with an epitope on endothelial cells. Janciauskiene et al. further confirmed these findings [2] and showed for the first time that the ATZ11 antibody recognizes a conformation-dependent epitope consisting of not only AAT molecules type PiZ but also of complexed non-Z-AAT and non-Z-AAT-elastase complexes [2]. Interestingly, ATZ11 staining of liver sinusoids is variable and reflects the hemodynamic alterations within the liver parenchyma [7], a phenomenon that may be related to an altered micro-vascular affinity to polymeric AAT/AAT-elastase complexes. Endothelial-bound polymeric AAT could also be demonstrated in normal and pathological lung tissue [8]. In the present study, we investigated the subcellular binding site of ATZ11 in endothelial cells and elucidated the role of VWF as a potential binding partner of ATZ11. Cytosolic VWF is accumulated within membrane-enclosed organelles known as Weibel-Palade bodies, which mainly contain densely packed tubular arrays of VWF and pro-peptides.

## Materials and Methods

### Ethics statement

Western blotting (WB) and native PAGE analyses of platelets and serum samples of a VWF-deficient patient were performed for hemostaseological diagnostics. However, no conclusion could be drawn for the individual whose samples were analyzed in this study. Consistent with the provisions and guidelines of the University ethics committee, the Institute of Pathology of Bonn Review Board Committee approved the participation of one healthy (non-Z) individual who contributed blood samples for WB analysis in this study. Written informed consent was given (as outlined in the PLOS consent form) to publish these case details.

Consistent with the directives on obtaining a general consent from patients for scientific research, the University ethics committee approved the retrospective analyses of two normal A. temporalis specimens of (non-Z) individuals and two liver biopsies of one (non-Z) individual and one patient carrying the heterozygous *PiMZ* mutation. All specimens were obtained from surgical excisions obtained for pathological diagnostics (‘Retrospective analysis of A. temporalis samples, and liver biopsies by immunohistochemistry’ (ref. 334/13)).

### Endothelial cells

Endothelial cells were extracted from umbilical cord veins and routinely cultured in RPMI 1640 supplemented with 10% fetal bovine serum, penicillin (100 units ml^−1^) and streptomycin (100 µg ml^−1^). Cells were cultured in humidified 5% CO_2_ and 95% O_2_ at 35°C. The endothelial monolayers were trypsinized for WB analysis.

### Immunoelectron microscopy

For immunoelectron microscopy, pellets or fragments of endothelial monolayers grown on membranes were immediately fixed by immersion with 3% paraformaldehyde and 0.1% glutaraldehyde in 0.1 M phosphate buffer (pH 7.6) for 2 h at room temperature [9]. After fixation, the fragments were washed in the same buffer, amidinized and embedded at progressively lower temperatures in Lowicryl K4M as previously described in Roth et al. [9]. Thin sections were cut with a diamond knife, mounted on 200-mesh nickel grids with carbon-coated formvar film, and processed for immunohistochemistry. Immunogold staining of the grids was performed using a modified protocol with avidin-biotin-complex according to Gee et al. [10]. Briefly, the staining procedure consisted of the primary antibody, biotinylated secondary antibody, streptavidin-biotinylated horseradish peroxidase complex and gold-conjugated anti-horseradish peroxidase antibody. Subsequently, the grids were counterstained with uranyl acetate (5 min) and lead acetate (45 s) and examined using a Phillips electron microscope (CM10).

Platelets were isolated and concentrated from the blood sample of the blood donor volunteer with *PiMM*-genotype (non-Z).

### Molecular analysis of the *AAT* genotype

Extraction of genomic DNA was performed using standard procedures. The quantity and length of the DNA molecules extracted from paraffin-embedded tissue samples were estimated using electrophoresis on a 1% agarose gel. AAT (Serpin A1) DNA was amplified by PCR using the following primers: S-Variants: forward 5′-GGT GCC TAT GAT GAA GCG TTT AGG C and reverse: 5′-AGG TGT GGG CAG CTT CTT GGT CA, Z-Variants: forward 5′-GTG TCC ACG TGA GCC TTG CTC and reverse: 5′-GTT TGT TGA ACT TGA CCT CGG. PCR was performed in 50-µl reactions that contained template DNA, 2 µM of each primer, 0.25 U Platinum Taq DNA polymerase (Invitrogen, Groningen, Netherlands), 5 µl of the corresponding reaction buffer, 200 µM of each dNTP, and sterile water. The MgCl_2_ concentration was 2.5 mM. The PCR conditions were as follows: initial denaturation at 94°C for 5 min; followed by 35 cycles with denaturation at 94°C for 60 sec, annealing at 60°C for 60 sec, and elongation at 72°C for 60 sec; and a final elongation at 72°C for 5 min. The PCR products were purified using Micro Spin columns (GE Healthcare, Munich, Germany). Template DNA concentrations for cycle sequencing were estimated using agarose gel electrophoresis. Bidirectional DNA sequencing was performed with the Big Dye Terminator Cycle Sequencing Ready Reaction Kit (Applied Biosystems, Weiterstadt, Germany) on all samples using the primers listed above. The cycle sequencing products were precipitated with 3 M sodium acetate and analyzed on an ABI PRISM 3130 capillary electrophoresis system (Applied Biosystems, Weiterstadt, Germany).

### Cell culture and transfection

Human embryonic kidney (HEK) cell lines (HEK293T and HEK293, DSMZ, Braunschweig, Germany) were used to express wild-type recombinant VWF (rVWF-WT) as previously described [11]. Briefly, cells were transiently transfected with 8 µg of rVWF-WT (Lipofectamine 2000; Life Technologies, CA, USA). Forty-eight hours after transfection of the HEK293 cells, the supernatants were collected and the cells were lysed for analysis of intracellular rVWF. RVWF secreted in the medium was concentrated on Amicon Centrifugal 50 K filter devices (Millipore, USA) to one-fourth of the original volume before subsequent analysis (Multimer analysis and Western blot). VWF antigen levels were measured using a particle-based turbidimetric assay (Siemens Healthcare, Germany). Briefly, small polystyrene particles attached to specific antibodies by covalent bonding were aggregated when mixed with samples containing rVWF antigen. Then, this aggregation was detected via the increase in turbidity.

### Confocal imaging

Transfected and non-transfected HEK293 cells were stained with ATZ11, anti-AAT and anti-VWF antibodies and secondary fluorochrome-labeled antibodies. Imaging was performed using Olympus Fluo View FV1000.

### Western blotting analysis (WB) of tissue cultures

The culture medium and HEK293 cell lysates were supplemented with NuPAGE LDS Sample Buffer (4×) (Invitrogen) and denatured at 95°C for 5 min. The protein standard (See Blue Plus 2, Invitrogen) and proteins were loaded on NuPAGE 4–12% Bis-Tris Gels (Invitrogen), placed in an Xcell Sure Lock Mini-Cell device (Invitrogen), filled with running buffer (Invitrogen) and separated at 170 V for 1.5 h. Proteins were transferred onto a polyvinylidene difluoride membrane (Roti-PVDF, Roth) using Xcell II Blot Module (Invitrogen) filled with NuPAGE Transfer Buffer. After blocking in 5% nonfat dry milk/TBST overnight, the membranes were incubated for 1 to 2 h using the following antibodies and dilutions: anti-human VWF (Dakopatts) 1∶800; ATZ11 (Wieslab AB) 1∶500. Next, the membranes were washed, incubated with horseradish peroxidase–conjugated secondary antibody (dilution 1∶2000; DAKO) and developed using enhanced chemiluminescence (GE Healthcare, Amersham).

### VWF Multimer analysis

SDS-agarose discontinuous gel electrophoresis was performed as previously described [12]. Briefly, medium resolution (1.6%) gel (LGT agarose type VII, Sigma, Munich, Germany) was prepared and the samples were diluted according to their VWF content in sample buffer. Electrophoresis was performed for ∼18 h at 55 V. After electrophoresis, the multimers were transferred onto nitrocellulose membrane by electroblotting using transfer buffer (0.05 M phosphate, pH 7.4 with 0.04 M SDS, without methanol). After transfer, nonspecific binding sites were blocked with low-fat milk. All of the following incubation and washing steps were performed in low-fat milk. The membrane was incubated overnight at a 1∶2000 dilution of polyclonal rabbit anti-human VWF antibody (Dako, Glostrup, Denmark) or 1∶250 dilution of monoclonal mouse anti-human AAT type PiZ antibody (Clone ATZ11). Detection was performed using chemiluminescence (Fluorchem™, Alpha Innotech Corp., San Leandro, California).

### WB analyses of platelets

The platelets of one healthy individual and VWF-deficient patients (negative control) were washed twice in PBS and clarified by centrifugation at 2500 rpm for 5 min. The cell pellets were re-suspended in a lysis mixture (50 mM TRIS-HCl, pH 8.0, 120 mM NaCl, 0,5% Nonidet P-40, 100 mM phenylmethylsulfonylfluoride, 1 µg ml^−1^ aprotinin) at 4°C for 30 min. The lysate was clarified by centrifugation at 14000 rpm for 15 min. The protein samples were mixed 1∶1 with Laemmli buffer (190 µl Laemmli, 10 µl DTT) and denatured at 95°C for 5 min and cooled on ice. The protein lysates were separated using SDS-PAGE. After equilibration with transfer buffer (BioRad, Hercules, California), the proteins were transferred from the gel onto nitrocellulose membranes using a semi-dry sandwich technique (0.1 A, 2 h). After blocking with human milk powder (100 ml TBS, 5 g human milk powder, 200 µl NaN_3_ at room temperature overnight), the membranes were incubated with primary antibodies (ATZ11 (1∶50), anti-VWF (1∶400)) overnight. After incubation with the goat anti-mouse/rabbit-conjugated secondary antibody for 2 h, the membranes were stained with an alkaline phosphatase conjugate kit (BioRad, Munich, Germany) and 5-bromo-4-chloro-3-indolylphosphate as the chromogen.

### Immunohistochemistry

Temporal arteries and liver samples were obtained from surgical specimens. Specimens were fixed in 4% neutral-buffered formalin and embedded in paraffin. After cutting, the sections were deparaffinized and developed in the immunostaining system TechMateTM 500 Plus (Dako). Blocking antibody was applied in increasing dilutions (1∶1000, 1∶500, 1∶100, 1∶50, 1∶10, and 1∶1) for 30 min at room temperature. Primary ATZ11 antibody (1∶50–1∶100), polyclonal anti-AAT (1∶1000–1∶30000), and polyclonal anti-VWF (1∶500–1∶2000) were added and allowed to react for 90 min at room temperature. Control slides were incubated with buffer or non-immunized mouse IgG (1∶1000, negative control). The secondary peroxidase-labeled antibody was applied and incubated for 30 min at room temperature. After washing, the sections were stained with DAB.

## Results

### Immunoelectron microscopic findings

**Figure 1:**
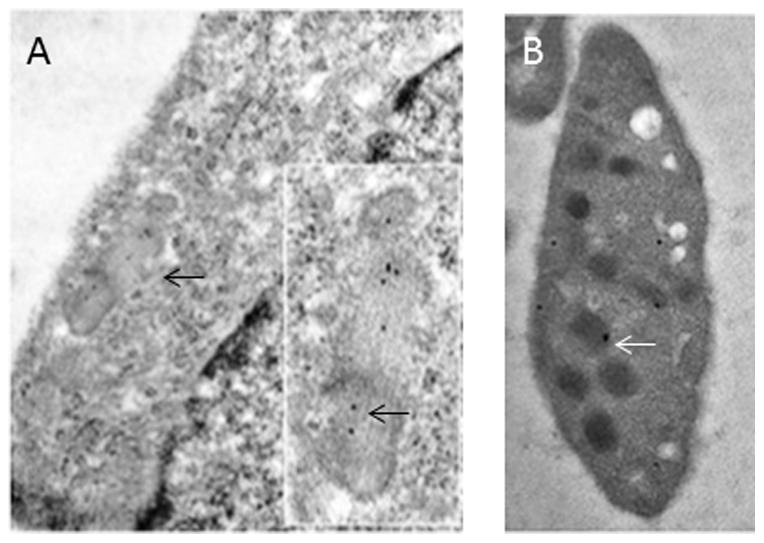
Subcellular distribution of ATZ11 antigen in the endothelium: (A) Immunoelectron microscopic labeling of ATZ11 in the vascular endothelium: A cutout of a HUVEC is shown. Within the cytoplasm (light zone that is silhouetted against the nucleus (right corner)), rod-shaped Weibel-Palade storage granules are located close to the cell's membrane (arrow). Gold particles (black dots) coupled to ATZ11 are located in a Weibel-Palade body (see inset) in human umbilical vein endothelial cells. (B) Platelets of a non-Z individual showed ATZ11-coupled gold particles in spherical α-granules within an electron-lucent zone (arrows).

On the basis of the observation that ATZ11 provides a specific cross-reaction with endothelial cells of hepatic arterioles and veins as reported in an earlier study [6], we aimed to reproduce this finding in cultured HUVECs using immunoelectron microscopy, a technique that allows precise sub-cellular localization of the target antigen. In cultured HUVECs, gold particles coupled to ATZ11 were concentrated on WPBs (**Fig. 1A**). Particles were found in the lumina and close to the surface and ends of WPB. Nuclei and cytoplasmic membranes were free of gold particles. Platelets of normal blood contained ATZ11-coupled gold particles in spherical α-granules within an electron-lucent zone (**Fig. 1B**). The distribution pattern of ATZ11-coupled gold particles within platelets and WPBs of HUVECS suggested that ATZ11 recognizes VWF molecules, a main constituent of both WPBs and α-granules.

### Immunocytochemical analysis of rVWF-WT-transfected HEK293 cells

To further confirm the potential VWF staining by ATZ11, we transfected HEK293 cells with recombinant VWF plasmid. Successful transfection was confirmed by the transfection of GFP-plasmid together with plasmids containing VWF cDNA, transfection was successful in approximately 60% of the HEK293 cells. RVWF production by HEK293 cells is outlined in table 1.

**Table 1:** Recombinant von Willebrand Factor Expression in four Transfection Experiments in HEK293 cell lines.

Transfections	Secreted rVWF (IU dL^−1^)	Intracellular rVWF (IU dL^−1^)
1	71	20
2	72	18
3	66	16
4	75	17
Average	71	17.8

rVFW – recombinant von Willebrand factor.

**Figure 2:**
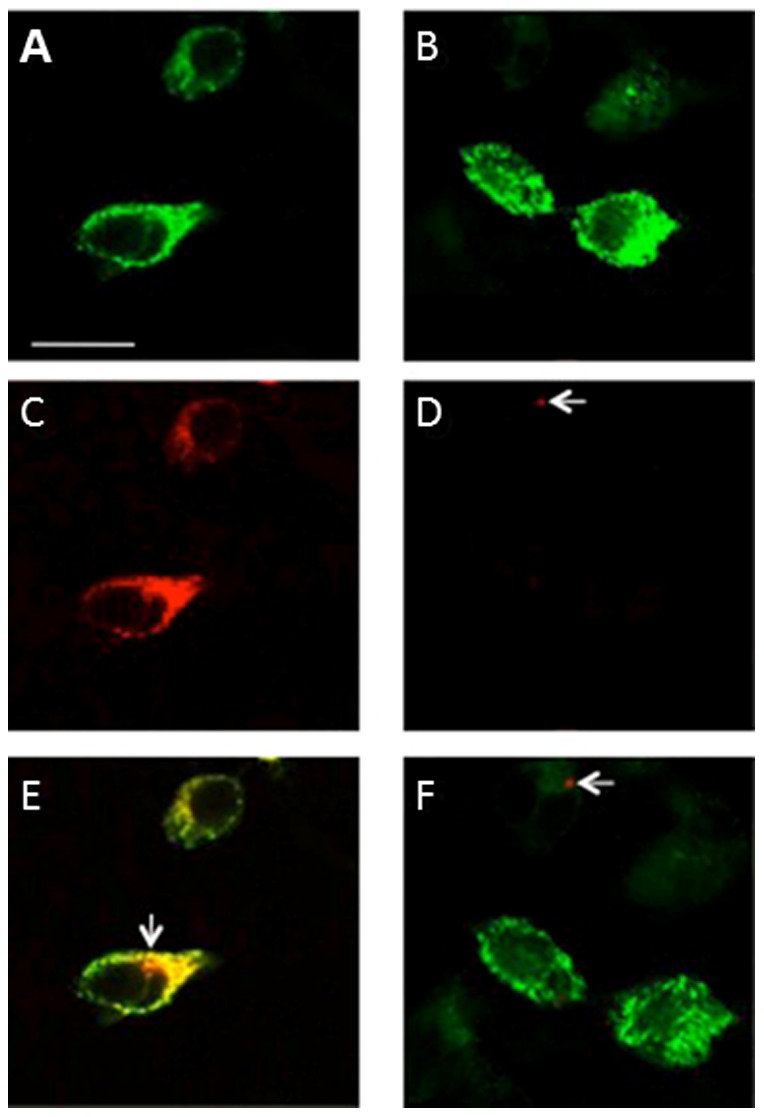
Confocal imaging of VWF-transfected HEK293 cells: Pseudo-Weibel-Palade-Body (pseudo-WPB) granules formed after transfection of HEK293 cells using recombinant wild-type VWF (rVWF-WT) constructs: (A, B) Pseudo-WPB granules are shown in green (anti-VWF staining). (C) The same intracellular structures are stained with ATZ11 (red). (D) Small dot-like signals of less than .25 µm were found in very few HEK293 cells stained with anti-AAT (arrow). (E) Merged images of anti-VWF and ATZ11 stains highlight the co-localization of the antibody-binding sites. At a single cell level, small dot-like positive signals were found in the ATZ11 reaction, which were not co-localized with VWF staining (arrow). (F) Merged images of anti-VWF staining and anti-AAT signals demonstrated that the dot-like anti-AAT positive signals were not associated with pseudo-WPBs (arrow). Scale bar = 10 µm.

Using confocal microscopy, we demonstrated that pseudo-WPB storage granules within transfected HEK293 cells stained positive with anti-VWF (**Fig. 2A, B**). Mock-transfected cells did not show any reaction with anti-VWF or secondary antibody (data not shown). Similarly, the same cells reacted with ATZ11 (**Fig. 2C**). In addition, non-transfected cells did not react with either ATZ11 or secondary antibody (data not shown). Importantly, in the anti-AAT reaction, we found scarce cell-associated positivity with a maximum size of less than .25 µm in only a few HEK293 cells. This reaction was observed in the mock-transfected and rVWF-WT-transfected cells (**Fig. 2D**). Merged images of anti-VWF- and ATZ11 staining highlighted the near complete co-localization of anti-VWF and ATZ11 binding within pseudo-WPBs (**Fig. 2E**). At a single cell level, small dot-like positive signals were found in the ATZ11-reaction, which were not co-localized with VWF-staining (**Fig. 2E** **, arrow**). Merged images of anti-AAT- and anti-VWF-staining demonstrated that the small dot-like anti-AAT positive structures were not localized with the VWF-molecules (**Fig. 2F**).

### Western blotting analysis of HEK293 cells and human serum samples

**Figure 3:**
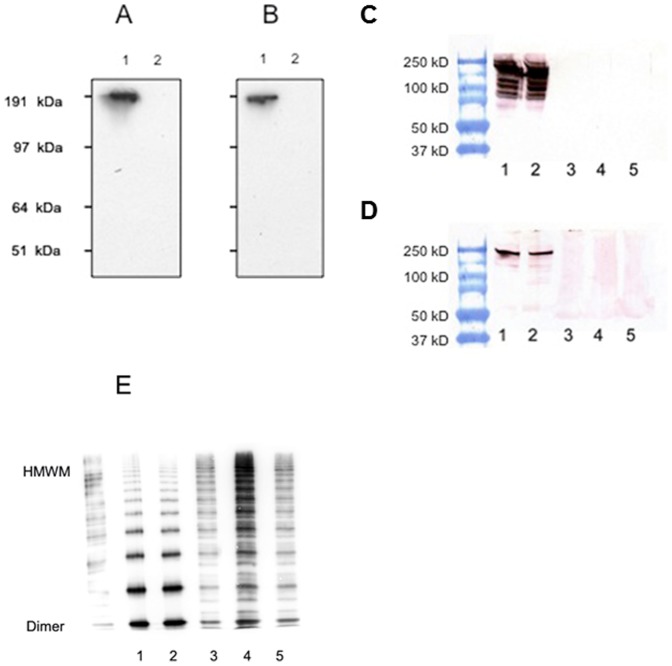
Protein-electrophoretic studies on VWF-transfected HEK293 cells and human serum samples: (A) **SDS-PAGE electrophoresis and subsequent western blotting (WB) and visualization using anti-VWF: (lane 1) cell lysates of recombinant wild-type VWF (rVWF-WT)-transfected HEK293 cells, (lane 2) mock-transfected HEK293 cells.** (B) SDS-PAGE electrophoresis and subsequent WB and visualization using ATZ11: (lane 1) of cell lysates of rVWF-WT-transfected HEK293 cells and (lane 2) mock-transfected HEK293 cells. A congruent single band of 225 kDa was detected in the VWF-transfected HEK293 cells using both anti-VWF (A) and ATZ11 (B). (C) SDS-PAGE electrophoresis and subsequent WB of human serum samples of a non-Z healthy individual (lanes 1–2) and of VWF-deficient patients (lanes 3–5) stained with anti-VWF. (D) SDS-PAGE electrophoresis and subsequent WB of serum samples of a non-Z healthy individual (lanes 1–2) and of VWF-deficient patients (lanes 3–5) stained with ATZ11. (E) Native PAGE electrophoresis and subsequent WB of a recombinant VWF (lanes 1–2) and serum samples of a non-Z healthy individual (lanes 3–5) stained with the anti-VWF antibody.

Our immunocytochemical findings were supported by additional protein-electrophoretic studies. We characterized the binding capacities of VWF and ATZ11 antibodies with intracellular and secreted rVWF-WT using WB. In the denatured proteinaceous content of cell lysates and conditioned medium of transfected HEK293 cells, we detected a congruent single band of 225 kDa using both anti-VWF (**Fig. 3A**, lane 1) and ATZ11 (**Fig. 3B**, lane 1). Culture medium and cell lysate obtained from mock-transfected HEK293 cells (non-transfected) served as an internal negative control (**Fig. 3A, B** lanes 2).

In the SDS-PAGE electrophoresis and subsequent WBs of serum samples, the anti-VWF antibody stained several bands between 120 kDa and 225 kDa (**Fig. 3C** **, lane 1, 2**). ATZ11 detected a single band of 225 kDa (**Fig. 3D** **, lane 1, 2**). We found that ATZ11 and anti-VWF stained denatured VWF molecules. Moreover, Anti-VWF also detected smaller VWF cleavage products (**Fig. 3C** **, lanes 1, 2**). However, neither antibody stained the blood samples of VWF-deficient patients (**Fig. 3C, D** **lanes 3–5**). Because SDS-PAGE results in denatured proteins, we inferred from these findings that ATZ11 detects the open VWF protein of 225 kDa.

Native PAGE-electrophoresis was performed on transfected HEK293 cells and serum samples. VWF multimers (550 kDa to 225 kDa), both recombinant and native, were devoid of ATZ11-labeling (data not shown), whereas anti-VWF distinctly recognized not only the recombinant multimers but also multimers of human serum samples and VWF cleavage products (**Fig. 3E**). We inferred from these findings that in contrast to cellular-bound rVWF-WT, native secreted rVWF-WT and human plasma VWF multimers cannot be identified using ATZ11.

### Immunohistochemical evaluation

**Figure 4:**
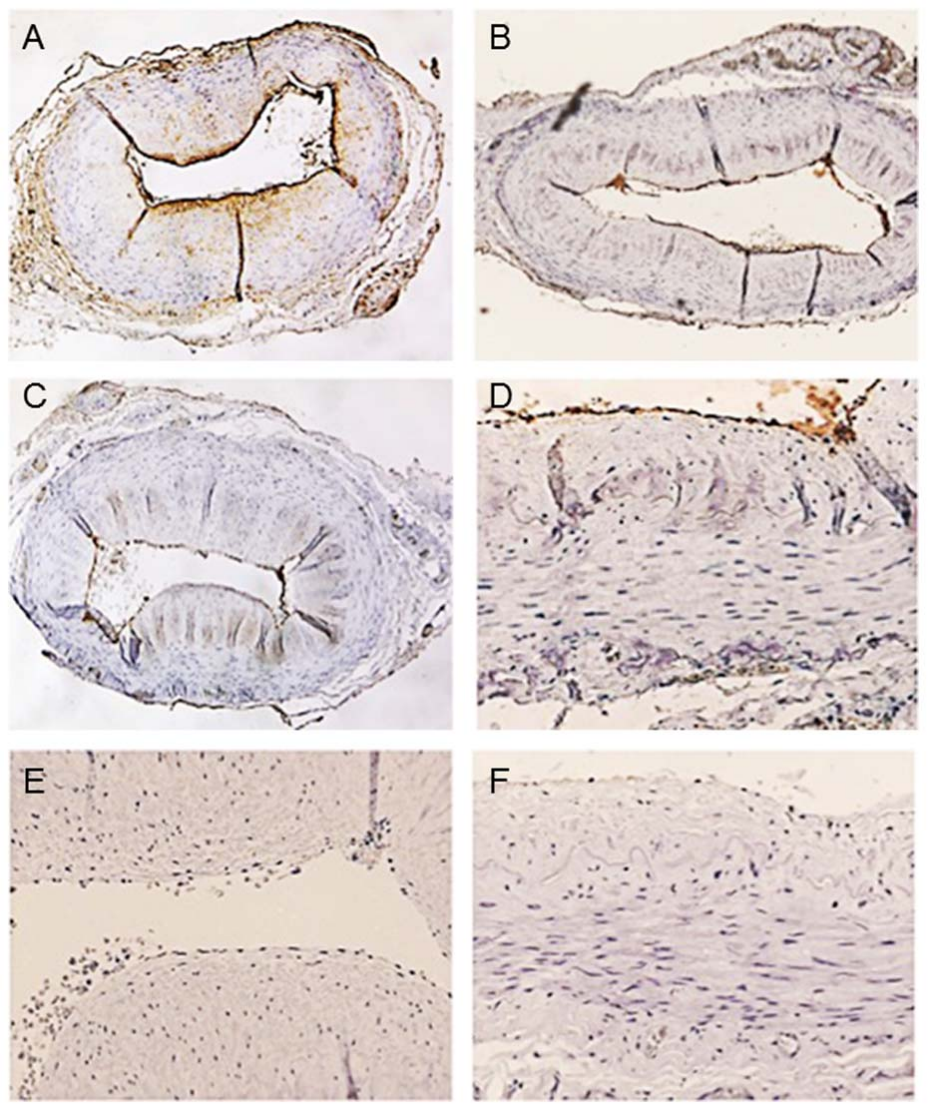
Comparative immunostaining of temporary artery specimens. (A) Localization of AAT in the temporal artery. Specimen stained with polyclonal anti-AAT (1∶5000) showed immunoreactivity on the endothelial surface and a gradient of presumably soluble AAT within the vessel wall. (B) ATZ11 (1∶100) showed a distinct staining of the endothelial layer. (C) The endothelial layer is distinctly stained using anti-VFW (1∶500). (D) After saturation with anti-AAT antibody (1∶10), ATZ11 labeled a thin endothelial layer. (E) Blockade with anti-VWF antibody (1∶10) abolished ATZ11 staining (1∶100) of the endothelial layer. (F) Sequential blockade with anti-AAT (1∶10) and anti-VWF (1∶10) completely abolished ATZ11 staining (1∶100).

Further immunohistochemical analyses revealed the specificity of VWF detection using ATZ11. First, temporal artery specimens were stained with ATZ11, anti-AAT, and anti-VWF in varying antibody concentrations. All three antibodies stained a thin endothelial layer (**Fig. 4A–C**). In addition, Anti-AAT staining displayed a gradient across the vessel wall, which presumably represented the diffusion of soluble AAT protein from the vascular lumen into the vessel wall [19] (**Fig. 4A**). To evaluate the capability of ATZ11 in marking VWF protein, blocking experiments were performed. In the first step, specimens were saturated with anti-AAT antibody in varying concentrations and subsequently stained with ATZ11. After complete saturation of AAT-antigens, ATZ11 still stained the thin endothelial layer (**Fig. 4D**). In the second step, anti-VWF was applied to block its specific binding sites prior to ATZ11 staining. The endothelial staining of ATZ11 disappeared (**Fig. 4E**). After sequential blockade was performed with anti-VWF and anti-AAT, the ATZ11 staining was negative (**Fig. 4F**).

In addition, the liver parenchyma of a *non-Z*-patient with chronic myeloproliferative disease (*PiMM*-genotype) was analyzed using ATZ11, anti-AAT, and anti-VWF antibodies. The liver sinusoids contained abundant megakaryocytes (MK). ATZ11, and anti-VWF antibodies brightly stained the MK cytoplasm and non-sinusoidal endothelium (see **Fig. S1**). However, the hepatocytes as well as granulocytes remained unstained. In contrast, anti-AAT staining of MK was very faint, whereas the granulocytes and hepatocytes were broadly stained. After blocking with anti-AAT antibody, ATZ11 still stained the MK and non-sinusoidal endothelium. However, blockade by increasing concentrations of anti-VWF antibody abolished ATZ11 staining of MK and non-sinusoidal endothelium similar to double blockade with anti-AAT and anti-VWF.

In the third approach, the liver tissue of a *Z*-individual (*PIMZ*-genotype) was obtained (see **Fig. S2**). Both, anti-AAT and ATZ11 stained the retained Z-AAT protein in hepatocytes. In addition, ATZ11 stained non-sinusoidal endothelial cells. Anti-VWF also stained non-sinusoidal endothelial cells, while the Z-AAT protein was negative.

After blocking with anti-AAT antibody, ATZ11 still stained non-sinusoidal endothelial cells. However, blockade with increasing concentrations of anti-VWF antibody abolished ATZ11 staining of non-sinusoidal endothelial cells, while Z-AAT-deposits remained stained. Double blockade with anti-AAT and anti-VWF completely abolished ATZ11 staining.

## Discussion

The ATZ11 antibody is a suitable marker for the immunohistochemical diagnosis of AAT-deficiency type PiZ in liver biopsies [4], [5], [6]. Several years ago, Janciauskiene et al. demonstrated that ATZ11-staining is not confined to Z-AAT-polymers. ATZ11 also reacts with non-Z-AAT-elastase complexes and complexed forms of non-Z-AAT [2]. They demonstrated that these binding characteristics do not lessen its diagnostic value in the detection of AAT-deficiency type PiZ because complexed forms of non-Z-AAT or non-Z-AAT-elastase complexes are not present in sufficient quantities to obtain a positive staining reaction by ATZ11 in ELISA [2]. As complexed forms of non-Z-AAT or non-Z-AAT-elastase complexes are very rare within the hydrophilic milieu of blood [13], we hypothesized that a target antigen might add to the strong endothelial staining reaction of ATZ11 [20], [21], [22], [23]. We proposed that in conjunction with the well-documented and frequently reproduced pathway mechanisms involving complex protein-protein interactions of the AAT molecule within human vascular bed [2], [3], [4], [8], [13], [14], [15], [16], our data may provide a straight-forward and new aspect to the understanding of ATZ11-antigen binding.

Our immunoelectron microscopic analysis strongly supports the notion that ATZ11, in addition to complexed forms of non-Z-AAT or non-Z-AAT-elastase complexes, stains VWF in WPBs of endothelial cells and α-granules of platelets. Subcellular distribution of ATZ11 reactivity was similar to that of VWF [17], [18].

Next, we aimed to reproduce this finding *in vitro* by transiently transfecting rVWF-WT in HEK293 cells. Our *in vitro* data clearly demonstrated that ATZ11 stained pseudo-WBPs in rVWF-WT-transfected HEK293 cells, whereas mock-transfected cells were negative using confocal fluorescent imaging. Anti-AAT-staining of rVWF-WT-transfected HEK293 cells was negative in pseudo-WPB, indicating that rVWF-WT binding was not mediated by AAT proteins. We hypothesized that the small dot-like anti-AAT reactions might reflect non-Z-AAT complexes within and in close proximity to HEK293 cells. These structures were also identified by ATZ11 (**Fig. 2E**, arrow). However, the vast majority of ATZ11 signals were congruent with anti-VWF reaction, indicating a reactivity of ATZ11 with the VWF protein. We affirmed this finding using SDS-PAGE and subsequent WB analysis.

In support of the latter, native PAGE electrophoresis and subsequent WB of recombinant as well as human VWF samples revealed that rVWF-WT and human VWF dimers and VWF multimers cannot be detected using ATZ11, in contrast to the denatured form of dehiscised VWF protein. This explains why ATZ11 signals are not observed in serum samples of non-Z individuals using ELISA, although VWF dimers and multimers are abundantly present in the serum.

In addition, further immunohistochemical blocking experiments clearly demonstrated that ATZ11 specifically cross-reacts with VWF in the vascular endothelium and MK in human tissue.

Taken together, this study identifies VWF as a novel target protein of ATZ11. Thus, ATZ11 may serve as a valuable diagnostic tool for the detection of endothelial cell proliferates and MKs in immunohistochemistry. In contrast, VWF dimers and multimers, the physiological state of VWF within human serum, were not identified using ATZ11.

## Supporting Information

Figure S1 **Comparative immunostaining of a liver biopsy of a non-Z patient with chronic myeloproliferative disorder and hepatic blood formation.** (A) Localization of AAT in liver tissue. Specimen stained with anti- AAT (1∶5000) shows immunoreactivity within hepatocytes, in intrasinosoidal megakaryocytes (MK), and portovenous endothelial cells. (B) ATZ11 (1∶100) distinctly decorated MKs and portovenous endothelial cells. (C) MKs and non-sinusoidal endothelial cells were stained by anti-VFW (1∶500) too. (D) After saturation with anti-AAT antibody (1∶10), ATZ11 (1∶100) still decorated MKs and portal venous endothelium. (E) Blockage anti-VWF antibody (1∶10), abolished ATZ11 (1∶100) staining of venous endothelial layer and MKs. (F) Sequential blockage with anti-AAT (1∶10) and anti-VWF (1∶10) totally abolished ATZ11 (1∶100) staining.
(PDF)

Figure S2 **Comparative immunostaining of a liver specimen of a Z patient (** ***PiMZ*** **-genotype) with liver cirrhosis.** (A) Localization of AAT in liver tissue. Specimen stained with polyclonal anti-AAT (1∶5000) shows immunoreactivity within hepatocytes. (B) ATZ11 (1∶100) distinctly decorated PiZ deposits within hepatocytes and the endothelial layer of portal veins. (C) The endothelial layer is stained by anti-VFW (1∶500) too. (D) After saturation with anti-AAT antibody (1∶10), ATZ11 still decorated portal venous endothelium, while PiZ deposits were negative. (E) Blockage with anti-VWF antibody (1∶10), abolished ATZ11 (1∶100) staining of endothelial layer, however hepatic PiZ deposits were decorated. (F) Sequential blockage with anti-AAT (1∶10) and anti-VWF (1∶10) totally abolished ATZ11 (1∶100) staining.
(PDF)
