# Supplementary figures and images for: ATZ11 Recognizes Not Only Z-α1-Antitrypsin-Polymers and Complexed Forms of Non-Z-α1-Antitrypsin but Also the von Willebrand Factor
Source: PLoS One. 2014 Mar 19;9(3):e91538. doi: 10.1371/journal.pone.0091538 (PMC3960128; doi:10.1371/journal.pone.0091538)

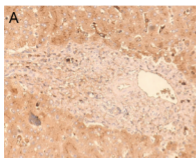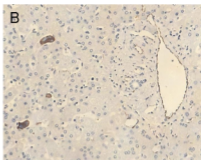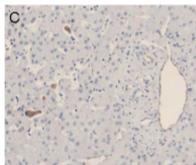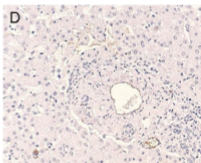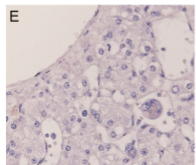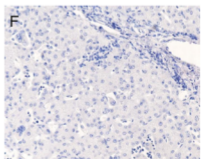

**Figure S1**

Supplement: Figure S1 — Comparative immunostaining of a liver biopsy of a non-Z patient with chronic myeloproliferative disorder and hepatic blood formation. (A) Localization of AAT in liver tissue. Specimen stained with anti- AAT (1∶5000) shows immunoreactivity within hepatocytes, in intrasinosoidal megakaryocytes (MK), and portovenous endothelial cells. (B) ATZ11 (1∶100) distinctly decorated MKs and portovenous endothelial cells. (C) MKs and non-sinusoidal endothelial cells were stained by anti-VFW (1∶500) too. (D) After saturation with anti-AAT antibody (1∶10), ATZ11 (1∶100) still decorated MKs and portal venous endothelium. (E) Blockage anti-VWF antibody (1∶10), abolished ATZ11 (1∶100) staining of venous endothelial layer and MKs. (F) Sequential blockage with anti-AAT (1∶10) and anti-VWF (1∶10) totally abolished ATZ11 (1∶100) staining. (PDF) [file pone.0091538.s001.pdf]

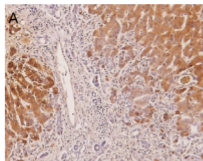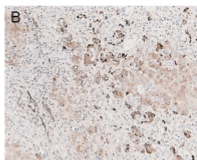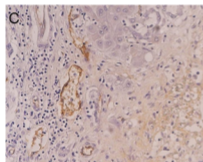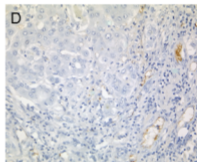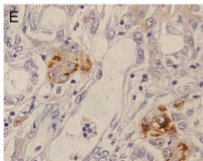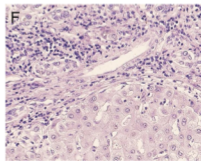

Figure S2

Supplement: Figure S2 — Comparative immunostaining of a liver specimen of a Z patient ( PiMZ -genotype) with liver cirrhosis. (A) Localization of AAT in liver tissue. Specimen stained with polyclonal anti-AAT (1∶5000) shows immunoreactivity within hepatocytes. (B) ATZ11 (1∶100) distinctly decorated PiZ deposits within hepatocytes and the endothelial layer of portal veins. (C) The endothelial layer is stained by anti-VFW (1∶500) too. (D) After saturation with anti-AAT antibody (1∶10), ATZ11 still decorated portal venous endothelium, while PiZ deposits were negative. (E) Blockage with anti-VWF antibody (1∶10), abolished ATZ11 (1∶100) staining of endothelial layer, however hepatic PiZ deposits were decorated. (F) Sequential blockage with anti-AAT (1∶10) and anti-VWF (1∶10) totally abolished ATZ11 (1∶100) staining. (PDF) [file pone.0091538.s002.pdf]
